# Translating facilitated multimodal online learning into effective person-centred practice for the person living with dementia among health care staff in Australia: an observational study

**DOI:** 10.1186/s12877-020-1417-3

**Published:** 2020-01-31

**Authors:** K. DeSouza, S. W. Pit, A. Moehead

**Affiliations:** 1 Dementia Care Competency & Training Network, Northern NSW Local Health District, LMB 11, Lismore, NSW 2480 Australia; 2 Western Sydney University, University Centre for Rural Health, 61 Uralba Street, Lismore, NSW 2480 Australia; 3 University of Sydney, University Centre for Rural Health, 61 Uralba Street, Lismore, 2480 NSW Australia

**Keywords:** Dementia, Facilitated, Knowledge transfer, Multimodal, Online learning, Person-centered care, Web-based

## Abstract

**Background:**

This paper aims to identify whether health care staff perceive a 12-week online facilitated, multimodal, person-centred care, dementia education program influences their knowledge, skills, behaviour and practice improvement activities in dementia care. In particular it will examine a dementia education program ‘Positive Approach to Care of the Older Person with Dementia’ (The Program).

**Methods:**

Three clusters of online questions were developed. Participants completed the first cluster at course completion (*N* = 1455;2013–2016). The second cluster was added into the 2015–2016 exit-surveys to measure clinical practice improvement (CPI) activities implementation (*N* = 520). Thirdly, all participants were invited to a 2018 follow-up survey (*N* = 343). The Program was also matched with key factors that are likely to result in effective online dementia education programs.

**Results:**

The Program had a 78% completion rate. At course completion (2013–2016, *N* = 1455), 62% felt that the online forums generated useful discussion and 92% thought their work would support implementing their new knowledge and skills. In 2015–16, participants (*N* = 520) reported that The Program had influenced their practice in terms of new knowledge (87%), understanding (87%), awareness (88%), and new ideas about delivering dementia care (80%). Almost all (95%) participants indicated they had changed ‘an aspect of their own professional practice’. Sixty-three percent had planned to develop a CPI activity. Of those (*N* = 310), 40% developed a new or improved tool and 21% planned to deliver education or create new resources. The most common CPI activities reported in the 2018 follow-up survey (*N* = 343) included education (49%) and role modelling of new behaviour (47%). Additionally, 75% indicated their CPI influenced their practice and had influenced patients (53%) and colleagues (53%). Fifty-seven percent reported their projects were sustained for 12 months or more.

**Conclusion:**

The *Positive Approach to Care of the Older Person with Dementia* education program can potentially improve training the dementia workforce. Participants perceived that a multimodal online platform facilitated by clinical champions influences knowledge transfer, skills and behaviour, encourages workplace CPI activities. Further effort could be directed towards empowering and supporting care staff on system, procedure and practice change and engaging management to translate training activities into practice.

## Background

Dementia affects almost 50 million people worldwide, and is predicted to increase to 131.5 million people by 2050 [1], resulting in the growing demand for a skilled and knowledgeable workforce. There is an urgent need for clinicians to have user friendly and accessible education, peer support, supervision and contact with dementia experts, especially for those in rural and remote regions. Online learning has the capacity to deliver flexible, accessible and cost-effective training platforms to a wide audience regardless of their setting or location [2].

### Impact of dementia and need for training

In 2015, about 46.8 million people globally were living with dementia. It is expected this number will reach 74.7 million in 2030 and 131.5 million in 2050. In high-income countries, this number will grow by 116% between 2015 and 2050 [3]. In 2018, the estimated cost of dementia in Australia is more than $15 billion [1]. Furthermore, people with dementia occupy up to one quarter of Australian hospital beds [4, 5] and account for 52% of all residents in residential aged care facilities [1].

The 2017 Australian National Safety and Quality Standards under Comprehensive Care Standard 5 highlight: “*People with cognitive impairment who are admitted to hospital are at a significantly increased risk of preventable complications such as falls, pressure injuries, delirium and failure to return to premorbid function, as well as adverse outcomes such as unexpected death, or early and unplanned entry into residential care… Although cognitive impairment is a common condition experienced by people in health service organisations, it is often not detected, or is dismissed or misdiagnosed….*. “ [6].

Staff working in acute hospitals report lack of knowledge, skills and confidence in caring for people with dementia [7]. In most countries, the low levels of awareness and training of healthcare staff [8] contribute to low rates of diagnosis [9] because dementia is often considered a normal part of ageing or is confused with delirium.

For those who are diagnosed, the lack of professional knowledge about treatment and care options may deny people access to optimal post-diagnostic care, treatment and support. Clinical benefits from early intervention and diagnosis take into account the right to a diagnosis, the potential for advanced care planning and the opportunity to optimise medical care including attention to physical comorbidities, better knowledge of available services, and timely delivery of additional support and care when the need should arise [10, 11]. Online learning can provide a platform for health professionals to access flexible education to improve awareness, knowledge and skills in delivering quality dementia care [12].

### Dementia care competency & training network (the network)

#### History

To address the need for improved education in dementia care, the *Dementia Care Competency & Training Network* (The Network) commenced in Australia in 2003, initially under a different name, *Acute Care Dementia Education & Training Resource.* This innovative online program, facilitated by dementia champions, was developed to advance the skills, knowledge and practice of clinicians in order to improve quality of life and care for people living with dementia. Since its inception, The Network has delivered high quality dementia education and resources to over 10,000 clinicians working within a variety of settings, including acute, community and residential aged care, across New South Wales (NSW).

The initial training package was commissioned by the NSW government in a response to identifying best practice for clinicians working with people living with dementia in acute care. Through collaboration, dementia experts created The Program which was trialled in several hospitals [13]. The initial series, of five modules, were delivered in paper format, CD ROM and PowerPoint presentations. Early expectations were that all newly appointed NSW Health Dementia Clinical Nurse Consultants (CNCs) were to implement the training state-wide with the focus on influencing a person-centred approach to clinical practice. In 2006, the paper versions were transformed into a web-based interactive online, clinician facilitated education, training and resource program. After a number of iterations, syllabus reviews and additional allocation of funding [14–16] the current Network emerged.

#### Online learning platform and dementia competencies

The Network provides a multifaceted education platform that assists clinicians across a variety of settings to meet the challenges of caring for the person living with dementia and meet professional obligations for lifelong learning. The Network is an online website comprising a Content Management System (CMS) and a Learning Management System (LMS) integrated into a single user experience which provides continuous membership, allowing ongoing access to resources, tools, clinical experts and forums. For the purpose of this paper the term participant/s and learner/s are interchangeable.

The Network allows participants to complete a self-assessment to identify their own perceived level of dementia competency. The Network is directed by a decision matrix consisting of identified domains of practice leading to three levels of dementia competency [17]. The assessment and development of these competencies enable the participant and the healthcare system to align compliance with the 2017 Australian National Safety and Quality Standards [6], in particular Comprehensive Care Standard 5. The Network has embedded these standards into the syllabus content across all courses. The contents and resources are affiliated with the dementia domains of practice [17], guiding an individualised learning pathway unique to the learner’s level of knowledge, skill and work setting. Health managers can monitor the clinician’s portfolio and body of evidence developed once achieving their clinical competency, which is reflected in their scope of practice.

#### Features of the network

The Network is free to NSW Health staff, our partners (e.g. residential aged care and general practice staff) and International Psychogeriatric Association (IPA) members. Interested participants are recruited from a number of sources e.g. local advertising, word of mouth and My Health Learning (NSW Health education platform). These individuals are invited to enroll in any of the five programs as start dates become available. Network trained facilitators support and encourage learners throughout the duration of courses, engaging in real time chats, responding to forum posts and grading progress. The Network staff provide ongoing support with IT, navigational, content, and log in assistance by way of an online help desk; supported through email or phone follow up.

Key features of The Network include:

- Five clinician facilitated learning courses: Positive Approach to Care of the Older Person with Dementia, Behavioural & Psychological Symptoms of Dementia, Positive Approach to Care of the Younger Person with Dementia, Positive Approach to Care of the Aboriginal Person with Dementia and Fundamentals of Person-Centred Care in Dementia.
- Highly interactive programs including: real time chats, forum posts, case studies and discussions, videos, tailored quizzes.
- A self-assessment resource measuring perceived knowledge and skills of dementia supported by a clinical mentor.
- Training modules on the competent delivery and interpretation of commonly used Cognitive Screening Tools.

As of March 2019, there were 5866 members in The Network and 97 facilitators. Fifteen NSW Local Health Districts are represented, including 3928 nurses, 714 allied health professionals, and 1224 other professionals, which embraces medical staff and non-regulated workers. Since 2013, on average 885 new members join each year. The annual delivery cost is estimated at $61 per person to access any course or resources within The Network. Additionally, 17,695 h of education were provided over a 10-month period in 2018. In 2018, the course completion rate across all courses was 76%.

Resources include a library of 730 + articles and a dementia community hub of blogs, Facebook, hot topics and ‘tea rooms’ for professional teams with an interest in dementia.

### Positive approach to the care of the older person with dementia (the program)

The 12-week course *Positive Approach to the Care of the Older Person with Dementia (The Program)* is one of the programs on The Network. The purpose of The Program is to advance the skills, knowledge and practice of clinicians focussing on the potential to change attitudes and behaviours, to implement person-centred practice and act as a catalyst in order to improve the quality of life and care for people living with dementia [18] http://dementiacare.health.nsw.gov.au/. The Program is facilitated by dementia clinical experts. Participants have access to their facilitator for the duration of The Program. Participants are expected to engage for a minimum of 2–3 h per week to complete all activities. The Program aims to increase participants’ knowledge and understanding of:

1. The need for a Person-Centred Approach [19]
2. Dementia and delirium
3. Why dementia is emerging as a critical issue for health care staff
4. Well-being and ill-being indicators for people with dementia
5. The concept and application of a Person-Centred Approach
6. Understanding the behavioural responses of people with dementia
7. The importance of involving the family/significant other in the health care setting for the person with dementia
8. The importance of multi-disciplinary team work in caring for the person with dementia.

**Box 1: Syllabus content of The Program** ***Positive Approach to the Care of the Older Person with Dementia***

**Table:** 

Week	Topic
1.	Navigating the Course
2.	General Introduction/Making Positive Change Happen
3.	Why use a Person-Centred Approach?
4.	Implementing a Person-Centred Approach
5.	Understanding How Dementia is Diagnosed
6.	Making Positive Change Happen Managing Dementia and Delirium in the Acute Care Setting
7.	Understanding and Managing Pain
8.	Restraint, Mobility and Falls
9.	Nutrition and the Effect of Fluid Balance
10.	Helping people with dementia cope with the challenges of swallowing, incontinence and bowel problems
11.	Discharge Planning and End of Life Planning
12.	Applying your Learning in the Workplace

### Key factors of effective online dementia training programs

In order to establish what features, lead to a functional web-based online dementia education program an extensive integrative literature review was undertaken [20]. Briefly, the review examined English language literature published between 2009 and 2018 from six electronic databases (Medline, Embase, CINAHL, Aushealth, Nursing@Ovid and Google Scholar). The review included 46 studies that focussed on health professionals and educational interventions that involved 1) web-based learning, on-line learning, internet-based education or Computer-Assisted Instruction; and 2) interactive facilitated or tutor supported education. Thematic analysis was used to review the literature and led to the creation of a framework of 14 key factors that are likely to create an effective online dementia learning environment (Table 1). The 14 features can be further classified into five key categories: Applicability, Attractiveness, Functionality, Learner Interaction and Implementation into Practice [8, 21–23, 27]. Kirkpatrick’s [12, 21, 25, 27, 32, 33] four core level model to evaluate training and education provision (i.e. learners’ reaction, extent of learning including knowledge, skills, confidence and attitudes, behaviour and practice change and outcomes on quality of patient care) aligns with the five key categories. The 14 key factors and consolidated five categories can be used to explore how they relate to online dementia training programs.

**Table 1:** Factors that are likely to create an effective online dementia learning environment and its correlation to the program *‘Positive Approach to Care of the Older Person with Dementia ’*

Factor	Examples of how each factor applies to the Program?
1. Self-directed/ self-paced learning [21, 22]	• Participants can work at their own pace, within their own time frame within the 12-week period.
2. Individualised, based on learner’s profile and background [12, 22]	• Program relates to all clinicians regardless of their role and experience.
	• Program measures different levels of competencies and tailors program content to these needs.
3. Interactive [8, 22]	• Participants take part in forums and real time chats where they can share ideas and work practices with other participants and interact with educational facilitators.
4. Multimodal [8, 22]	• Various forms of communication are used including the use of videos, virtual tours, animations, quizzes, case studies and the opportunity for discussions.
5. Flexibility [2, 23, 24]	• The program is flexible in that it can be delivered online, face to face or a combination of both. The program is flexible in delivery, accessibility and if people can’t attend the forums there are alternatives to completing the assessment tasks.
6. Accessible [2, 23, 24]	• The Program is available 24/7, 7 days a week, from anywhere, provided the person has a computer / tablet / device with access to the internet and is provided free of charge to the participant.
7. Consistency of information, repetition, reinforcement [8, 23]	• The program provides reliable, current, standardised syllabus content, offering a consistent teaching tool for facilitators and exposes the participant to evidence-based learning. Content is repeated and reinforced in multiple ways, such as quizzes.
	• Measures are in place that ensure that progression through the course only occurs when the participant achieves an 80% pass. If this mark is not achieved, they are able to repeat the lesson to help solidify the information learnt. Alternatively, the participant can liaise with their facilitator in regards to areas that need extra support.
8. Cost effective & good value for investment both for the learner and the system [2, 8]	• The program provides equitable access to all participants which benefits their learning path. It provides Continuing Professional Development towards their AHPRA registration needs and at the same time improves the care they deliver to the person living with dementia.
	• The Program reduces the rural / remote divide for clinicians through the accessibility and cost effectiveness of the program.
	• This benefits the health system as it provides a skilled and knowledgeable workforce through a consistent knowledge base.
9. Measures using questionnaires / feedback / surveys of satisfaction [7, 8, 23, 25, 26]	• Numerous opportunities are provided throughout the program to provide feedback and satisfaction on the learning experience.
	• Feedback is collected anonymously in order to encourage participants to provide honest responses.
10. Provides equitable engagement [21, 26, 27]	• All learners, regardless of role, are encouraged to participate in the program and have equal access to the facilitator, peers and resources.
11. Facilitated / access to instructor / mentored [22, 23, 27, 28]	• The program is facilitated by a clinical expert. The facilitator is there to support and encourage the learner and can be contacted anytime by the participants.
	• The network team is available to assist and support the facilitator with technical support and trouble shooting.
	• The program provides a supportive platform for facilitators.
12. Nurtures critical thinking, reflection & applicability [8, 26, 28, 29]	• This is achieved through the online chats, case studies and posting to the forum. Participants are encouraged to share barriers and positive outcomes they encounter in practice.
13. Establishment of a learning community [12, 21, 27, 28]	• Participants remain lifetime members of the network. Opportunities to interact on completion of the program continues through Facebook, forums, tea rooms, the newsletters and the monthly hot topic.
14. Ability for translation into practice [2, 27, 30, 31]	• Participants are encouraged to develop a quality improvement activity in their workplace that will improve the life of the person living with dementia.

### Study aims

This paper aims to explore whether health care staff perceive a facilitated, multimodal online dementia education program that focuses on person-centered care, influences their dementia knowledge, knowledge transfer, skills, behaviour and practice improvement activities in dementia care.

## Methods

Two main methods were used. Firstly, the design and delivery of The Program components were compared with factors derived from the literature [20] that are likely to create an effective online dementia learning environment. Secondly, a cross-sectional analysis was conducted using the feedback surveys that were completed by learners on completion of the 12-week online program Positive Approach to Care of the Older Person with Dementia over the period 2013–2016.

### Key factors of effective online dementia training program and its application to the program

Based on the integrative literature review [20], two steps were undertaken:

1. The design and delivery of The Program components were compared with the framework and its 14 factors that are likely to create an effective online dementia learning environment and how they are applied to The Program. (See Table 1 for 14 key factors)
2. The 14 factors were further consolidated into five categories which were used to analyse the questions from the feedback surveys in the cross-sectional analyses. (See Table 2 for five categories).

**Table 2:** Learners’ perspectives on network characteristics, 2013–2016, *N* = 1455

Domain: applicability	%	Domain: attractiveness	%	Domain: functionality	%	Domain: learner interaction	%	Domain: implementation into practice	%
To what extent was the course manual, videos and resources useful to you?		Did you find creating a Gravatar:		If you used the help- desk did you find it		Did you feel the forums generated useful discussion?		Extent work will support implementing new knowledge and skills	
Extremely	33	Extremely easy	3	Extremely useful	11	Yes	62	Great extent	46
Very	47	Very easy	21	Very useful	18	Sometimes	34	Some extent	46
Moderately	15	Moderately easy	37	Moderately useful	4	No	4	Little extent	6
Slightly	3	Slightly easy	10	Slightly useful	1			No extent	1
Not at all	2	Not at all easy	16	Not at all useful	1				
Not applicable	1	Did not use	13	Not applicable	65				
Did you find the content:		How visually appealing was the website		Helpdesk solution provided in timely manner?		Were the chats accessible?		Extent learner committed to implementing new practices?^a^	
Extremely easy	8	Extremely	20	Problem solved timely	22	Yes	75	Great extent	77
Very easy	28	Very	58	Problem solved > 24 h	7	Sometimes	22	Some extent	22
Moderately easy	54	Moderately	20	Problem not solved	1	No	4	Little extent	1
Slightly easy	9	Slightly	2	Not applicable	71			No extent	0
Not at all easy	2	Not at all	0						
To what extent did you already know the information presented?				Did you find navigating he website		Did you feel supported by your facilitator?^a^			
Great extent	11			Extremely easy	15	Extremely supported	39		
Some extent	73			Very easy	44	Very supported	46		
Little extent	15			Moderately easy	32	Moderately supported	12		
No extent	1			Slightly easy	7	Slightly supported	3		
				Not at all easy	2	Not at all supported	1		
To what extent were you able to understand the information?				Did you find joining the website:					
Great extent	87			Extremely easy	12				
Some extent	13			Very easy	39				
Little extent	0			Moderately easy	37				
No extent	0			Slightly easy	8				
				Not at all easy	5				

^a^ Data only available for 2013 and 2014, *N* = 935, including a mix of 19 open and closed questions. 2015–2016 included a mix of 71 open and closed questions

### Cross-sectional study

#### Procedure, participants, data collection

All participants successfully completing The Program are required to complete a de-identified online Moodle feedback survey as a mandatory component to completion. The feedback questionnaires from participants for the period 2013–2016 were selected for inclusion in the analysis. Only de-identified numbers were used to analyse the quantitative and qualitative data.

Comprehensive online data collection about participant’s feedback started in 2013. Data was obtained from three main sources and consisted of 71 questions using a mix of closed and open-ended questions. The 2013–2014 data included 2 open-ended questions with the revised questionnaires in 2015–2016 including 19 open-ended questions. Depending on the yes/ no response to the questions, participants were asked to add a written comment. Examples of the open-ended questions were;

- *Have you changed your beliefs or thinking about a particular approach or procedure? You answered yes. Please provide an example of how your beliefs/thinking about a particular approach or procedure has changed.*
- *Have you educated or informed a resident, patient, client or carer? You answered yes. Please provide an example where you have educated or informed a resident, patient, client or carer.*

The three main data sources were:

1. *Exit Survey 2013–2016 (N = 1455):* Upon completion of The Program questions focused broadly on: professional background, ease of navigation, syllabus content, learner support, applicability to clinical practice and transferability of new knowledge to the clinical setting [23]. On the question of: ‘*How will you translate your learnings into clinical practice’* there were 824 written responses included in the 2013–2014 data and for the 2015–2016 period 309 responses. However, given there were many opportunities in the 2015–2016 surveys to include written comments the most comments received were 541 responses to the question ‘*Have you changed an aspect of your own personal professional practice?*
2. *Exit Survey 2015–2016 (N = 520):* Throughout 2015, the exit survey questions were updated and expanded to include participant reflections on how they intended to translate learning into practice (*N* = 520). Firstly, additional questions focussed on how often any information or skills gained from The Program influenced participant’s clinical practices in a typical week in terms of new knowledge, increased understanding, raised awareness, attitude change, and new ideas about delivering care. Secondly, new questions focussed on self-reported behaviour change that occurred throughout the program through change in their own practice and their influence on others and their intention to upskill. Thirdly, questions were asked about future intentions around quality improvement projects.
3. *Follow Up Survey 2018 (N = 343):* An anonymous online survey was conducted in 2018 to evaluate how past participants had translated the knowledge, behaviour and skills gained from completing the course, into their workplace, and if clinical practice had been sustained. Unfortunately, some of the 2013–2016 participants were automatically un-enrolled due to a configuration setting within The Program, as such data was only available for 922 participants. Participants were asked about what project/s or activities they had intended to initiate, whether they had initiated them and whether the implementation had been sustained over time. These results formed the measure for whether their learning had been translated into practice overtime.

#### Data analysis

Statistical and descriptive analysis was performed using SAS Version 9.3 (SAS Institute, Cary, NC, USA).

An inductive qualitative analysis was used to analyse open-ended responses including the reasons for not completing a workplace project. Initially all answers were read to identify categories by the authors. Once consensus was reached on categories an initial count and analysis was conducted. This was then further trialed and refined in a second cycle of coding. The follow up coding process was carried out by the authors. The same qualitative process was undertaken to analyse participants’ experiences of how they would translate their learning into person centered-care for those living with dementia, in the context of their workplace. Excel and Word were used to organise the qualitative data.

## Results

### The network

The results of the surveys of 1455 participants who completed the 12-week course *Positive Approach to the Care of the Older Person with Dementia (The Program)* can be found in Tables 1, 2, 3, 4, 5 and 6.

**Table 3:** Learners’ perspectives on how the Program has influenced their knowledge, awareness, beliefs and attitudes on how to improve care for people with dementia, 2015–2016, *N* = 520

How often in your typical work week has any information or skills gained from the course influenced your practice in the following way:	Almost always % (n)	Frequently % (n)	Occasionally %(n)	Rarely %(n)
Has it given you **new knowledge or information** about how to care for residents/clients/patients	37 (191)	50 (261)	13 (65)	1(3)
Helped **you understand** the way you deliver care for residents/ clients/patients	34(177)	52(271)	13(65)	1(7)
Raised your **awareness** about new ways to care for residents/clients/patients	34 (176)	53(277)	12(63)	1(4)
Helped to **change your mind** about how to care for residents/clients / patients	34(176)	45(233)	19(96)	3(15)
Given you **new ideas** about how to care for residents/clients/patients	31(161)	49(255)	19(98)	1(6)

**Table 4:** Learners’ perspectives on how the program has influenced their behavior through change in their practice, influencing others and their intention to further up-skill. 2015–2016, *N* = 520

	n	%
Have you changed:		
An aspect of your own personal professional practice?	492	95
A practice or routine on your “unit” or in your workplace?	285	54
A procedure, technique or other intervention?	249	48
Your beliefs or thinking about a particular approach or procedure?	442	85
Have you informed or educated:		
A resident, patient or carer	400	77
Another member if staff	399	77
A member of the public?	183	35
Types of education to staff		
Face to face	365	70
Brochure	27	5
Poster	5	1
Conference	7	1
In-service	43	8
PowerPoint	17	3
Other	42	8
Types of education to the public		
Face to face	160	31
Brochure	23	4
Poster	7	1
Conference	2	0
In-service	7	1
PowerPoint	5	1
Other	19	4
Have you supported/assisted another staff member to make a change to their own practice?	338	65
Have you encouraged or supported a patient /client / resident / carer / member of the public to make a change in the way they understand dementia?	405	78
Have you created a new policy or guideline to support a new practice or procedure around dementia or delirium?	42	8
Recommend course to others	519	100
Intend to take:		
Further courses within dementia training network	459	88
Further studies in the specialty of dementia	384	74
Clinical care competencies	199	38
Are you planning to develop a Clinical Practice Improvement Project?	328	63

**Table 5:** Learners’ report of type of project intended to develop. 2015–2016, *N* = 310

Type of project:	n	%
New / improved tools	123	40%
- TOP 5 (clinician/ carer communication tool)	64	21%
- Creation of documentation to support family	24	8%
- Checklist	17	5%
- Assessment	6	2%
- Flip chart	1	0%
Delivery of education	66	21%
- In-service	14	5%
- Confused Hospitalised Older Persons (CHOPS)	1	0%
Resources/ information	56	18%
- Resource folder	23	7%
- Brochure	18	6%
- Booklet	3	1%
- Signage	1	0%
Undecided	22	7%
Research	14	5%
Policy	11	4%
Role modelling	9	3%
Other	6	2%
Promotion	3	1%
Total	310	100%

**Table 6:** Learner characteristics and analysis of implementation activities, *N* = 343^a^

	N	%
Discipline (*n* = 343)		
Nursing	250	73
Allied health	57	17
Other	36	10
Type of activities that were translated into practice due to learning (*n* = 337): ^b^		
Delivery of education	165	49
Role modelling of new behaviour	157	47
Promotion of the Program	128	38
Diversional activities *(eg photo album)*	67	20
Policy	36	11
Other	33	10
Brochure	16	5
What impact did the activity have on care provided in the workplace? (*n* = 287)		
Low	47	16
Medium	153	53
High	87	30
Has the activity impacted any of the following:		
Yourself	254	75
Colleagues	179	53
Patients	179	53
Carers	137	40
The workplace	99	29
System	28	8
Did the Program provide you with new knowledge and resources to (*N* = 343)		
- Provide improved care for people with dementia?	335	98
- Conduct a CPI project / activity	272	79
Has the CPI activity been sustained (*n* = 224)?		
Yes	179	80
No	45	20
How long has the CPI activity been sustained for? (*n* = 166)		
< 3 months	24	15
3–6 months	28	17
6–9 months	19	11
> 12 months	95	57
Reasons for not implementing an activity^c^: (*n* = 176)		
Lack of time	111	63
Workplace competing demands	99	56
Lack of workplace resources	34	19
Lack of management support	31	18
Limited support from colleagues	30	17
Do not feel I am in a position to influence change	29	16

^a^ Some missing data. ^b^Some participants undertook multiple CPI projects. ^c^ Multiple responses possible

### Key factors of effective online dementia training program and its application to the program

Table 1 outlines the 14 factors that are likely to create an effective online dementia learning environment and how they correlate to the Program*.*

### Cross-sectional analyses

Annual survey completion rates were stable across the 4 years between 2013 and 2016 *N* = 530 (78%), *N* = 405 (76%), *N* = 251 (78%) and *N* = 269 (72%) respectively. The majority of the 1455 participants were from a nursing background (*n* = 1048; 72%), followed by allied health (*n* = 218; 15%), management (*n* = 73; 5%), clinical support staff (*n* = 58; 4%) or other (*n* = 58; 4%).

#### Program characteristics

Table 2 demonstrates the learner’s perspectives on the five key categories [22] that lead to a functional web-based online education dementia program derived from Exit Survey 2013–2016 (*N* = 1455):

1. *Applicability:* (In this context it is defined as delivering an authentic learning experience via case studies etc. [22]). Overall the Program was applicable to the participants. Eighty percent (*N* = 1164) indicated The Program materials were ‘extremely ‘useful’ to ‘very useful’ and 54% (*N* = 786) perceived the content to be moderately easy. Eleven percent (*n* = 160) reported that they already understood the information to a ‘great extent’, and 73% (*n* = 1062) reported that they already knew the information to ‘some extent’. Eighty-seven percent (*n* = 1266) thought that they were able to understand the information to a ‘great extent.’
2. *Attractiveness****:*** Only a quarter (*n* = 364) thought that creating a Gravatar (an image that appears beside your name) was ‘extremely’ or ‘very easy’. Reasons for not using a Gravatar mainly centered on *‘did not want to’* or not knowing what a Gravatar was: *‘I am not sure what the Gravatar is’.* Seventy-eight percent (*n* = 1135) of participants found the website visually ‘extremely’ or ‘very’ appealing.
3. *Functionality****:*** The majority of participants did not use the helpdesk 65% (*n* = 786). Fifty-nine percent (*n* = 858) of participants found navigating the website ‘extremely easy’ 15% (*n* = 218) to ‘very easy’ 44% (*n* = 640). Similarly, 12% (*n* = 175) found joining the website ‘extremely easy’ and 39% (*n* = 567) found it ‘very easy’. Nonetheless, about one third (*n* = 485) reported they only found it ‘moderately easy’ to navigate the website or to join the website.
4. *Participant interaction****:*** Participant interaction through the forums was perceived to generate useful discussion 62% (*n* = 902) by participants, 75% (*n* = 1091) indicated the chats were accessible and 85% (*n* = 1237) felt ‘extremely supported’ or ‘very supported’ by their facilitators. Among the 22% (*n* = 320) of participants that reported that chats were only ‘sometimes’ accessible, some reported that ‘*the content was not deep enough’* or ‘*off topic*’ whilst others reported being unable to attend at allocated times. Others perceived the chats to deepen learning whereas for others the group dynamics hindered progress:
  *➢ ‘Good to have perspectives from different professions and work environments’ (ID-402)*
  *➢ ‘At times the chats would go in a direction that I did not feel I could contribute to. Sometimes my comments were missed by the group because of the number of people posting at the same time’ (ID-271)*
5. *Implementation into practice:* About three out of four learners 77% (*n* = 720) were committed to a ‘great extent’ to implementing new practices. Forty-six percent (*n* = 669) of the learners indicated that their work environment would support them to implement their new knowledge and skills to a *great extent*, a further 46% (*n* = 669) thought they would be supported to *some extent,* while only 6% (*n* = 88) of learners reporting their workplace would support them only to *a little extent.*

#### Translation into practice: change in knowledge, beliefs and attitudes

Table 3 outlines how often information or skills gained from The Program influenced participant’s practices in a typical week and was derived from Exit Survey 2015–2016 (*N* = 520). More than 80% (*n* = 416) thought that the information or skills gained from The Program influenced their practice ‘almost always’ or frequently in terms of new knowledge, understanding, awareness, changing their mind about how to care for their patients, and new ideas about delivering care. In the words of one participant:

- *➢ ‘I have changed the way I interact with, listen to and encourage participation into activities.’* (ID-118)

#### Translation into practice: behavioural change

Table 4 implies the majority of participants changed ‘an aspect of their own professional practice’ 95% (*n* = 494) or had changed their ‘beliefs or thinking about a particular approach or procedure’ 85% (*n* = 442). On the contrary, only half of participants had changed ‘a practice or routine in their workplace’ 54% (*n* = 281) or ‘a procedure/technique or other intervention’ 48% (*n* = 250), suggesting it is easier to change your own practice than to change shared standard work practices. However, it also appeared that participants were able to change co-workers individual work practices given the majority of learners had supported other staff to change their practice 65% (*n* = 338) and a large proportion had also informed or educated other staff members 77% (*n* = 400). One hundred percent (*n* = 520) of participants would recommend The Program to other staff members. Most learners felt they had influenced their clients or the public with 78% (*n* = 406) reporting that they had supported their clients/a carer or a member of the public to change the way they understand dementia. More specifically, most learners reported they had informed or educated a resident/patient or carer 77% (*n* = 400), whilst only one third had informed or educated a member of the public 35% (*n* = 182). Face-to-face education 70% (*n* = 364) and providing in-service to staff 8% (*n* = 42) were the most frequent actions taken to inform or educate staff. The most frequent actions taken by learners to educate the public were, face-to-face education 31% (*n* = 161) and handing out of brochures 4% (*n* = 21).

Only 8% (n = 42) of participants created a new guideline or policy to support a new practice or procedure around dementia or delirium at the time of completing the survey. A high proportion of participants indicated they would also undertake further courses 88% (*n* = 458) or further studies in dementia 74% (*n* = 385). However, only 38% (*n* = 198) suggested they would complete further clinical care competencies it is worth noting that around 50% (*n* = 260) did not know what the clinical care competencies were.

From the learner’s perspective:

- *➢* ‘*What I have found most valuable in this course is how ‘person-centred care’ as a concept has been articulated and translated to care of people with dementia. Prior to this course, I think I really feared dementia, probably out of ignorance. I have been delighted to learn how to promote and maintain ‘personhood’ against the challenges of the impact of this disease.’ (ID-111)*

#### Translation into practice: clinical practice improvement (CPI) project / activity intentions 2015–2016

Based on the Exit Survey 2015–2016 data, 63% (*n* = 328) (Table 4) of participants indicated they were planning to develop a CPI project / activity. Table 5 includes learners’ response to an open-ended question about the type of project they intended to develop (*n* = 310). Of those, 40% (*n* = 123) planned to develop a new or improved tool, with the majority 21% (*n* = 64) reporting implementation of TOP 5 [34]. TOP 5 is a communication tool for staff. Staff engage with relatives of people living with dementia and identify their top five tips on how best to communicate and work with the person living with dementia to enable person-centred care. Top 5 was followed by involving family 8% (*n* = 24); Participants proposed the initiation of a variety of documents in the workplace that family and carers would complete as a ‘getting to know me tool’. Twenty-one percent (*n* = 64) reported their intention was to focus on the delivery of education and 18% (*n* = 56) on the delivery of resources / information.

#### Translation into practice: clinical practice improvement (CPI) projects / activities implementation 2018

The response rate for the 2018 follow-up survey was 37% (*n* = 343/922). Of those who responded, the majority were nurses (Table 6). The most common activities that were implemented into practice were education 49% (*n* = 165) and role modelling of new behaviour 47% (*n* = 157). Thirty percent (*n* = 87) of participants indicated that the projects had a high impact on the care provided and 53% (*n* = 153) thought it had a medium impact. Only 8% (*n* = 28) thought the activity had influenced systems, but 75% (*n* = 254) indicated the project influenced their practice, 53% (*n* = 179) thought it had influenced patients and colleagues, 40% (*n* = 137) thought it had influenced carers and one third (*n* = 99) of participants responded that The Program had influenced their workplace. Of those who reported on the sustainability of their project, 28% (*n* = 47) reported their project was continued for up to 9 months and 57% (*n* = 95) of projects continue to be sustained for a period of 12 months or more. Overwhelmingly, the majority of participants responded that The Program had improved care for people with dementia and 79% (*n* = 272) reported they had conducted a CPI project / activity.

Of those who did not implement projects, 63% (*n* = 111) reported lack of time and 56% (*N* = 99) reported competing workplace demands as obstacles.

## Discussion

This study suggests that a facilitated multimodal online program may contribute to a more skilled healthcare workforce when person-centred care knowledge is translated to the practice environment. Furthermore, from the learners’ perspective, the results support a concept that online learning programs can be designed not just in terms of modules and content but on an entire educational experience as this may potentially improve clinical practice, critical thinking and reflection, with a desired outcome that may potentially result in the best possible provision of care for people living with dementia [2, 12, 28].

### Practice improvement

Constructivism learning fosters the capability to manipulate and interpret information as well as increasing confidence in meeting competency [35, 36]. Participants were exposed to a learning environment that invited participants to use information gained from the course and then interpret that information regarding dementia care in their own working environment (eg through the weekly chat-room discussions). The validity of constructivism learning suggests it could be useful in guiding the design of learning experiences, in particular electronic, with the possibility of it providing positive outcomes such as the acquiring of knowledge [28, 37]. Constructivism learning is reflected in the value of self-reported feedback as self-reported feedback suggests learning can result from activity, critical reflection and self-organisation. Furthermore, constructivism learning occurs through the learner being able to use their existing knowledge and life experiences. Participants were encouraged to construct their reality through applying this to case scenarios, forum posts, chat rooms and questions embedded throughout The Program. Therefore, the theory of constructivism supports the adult learning principles by encouraging the learner to contribute and construct reality of their personal and clinical experience and prior knowledge of dementia to the learning journey. Many of the learners discussed their experiences of dementia throughout the course and during forum posts and real live chats. This will often influence how they translate the learning into practice.

It appears that forum discussions and chats provide an opportunity for learners to interact with other participants, facilitators and clinical champions and at the same time reflect on their current practice. Learners overall reported that the support and motivation from the learning community provided encouragement to implement new practices and knowledge in the work environment. One participant commented: *The course has been very informative and challenges thinking about clinical practice. The course convener was supportive and kept the chat forums progressing and on topic. (ID-276).*

The majority of participants reported that the skills, knowledge, understanding and awareness gained influenced their professional practice by changing their beliefs and thinking. Changing the work practice of co-workers and education of carers and residents was also reported as an achievement which may be an indication of the efficacy of collaborative learning and suggests a flow on effect of learning beyond the learner. However, only 46% of the learners indicated that their work environment would support them to implement their new knowledge and skills to a great extent, whilst 46% of learners felt their workplace would support them to some extent or only to a little extent (6%). It is important to have a supportive workplace to implement new knowledge and skills. For example, the learning experience provides an additional opportunity for the learner to undertake a CPI project / activity as a way of applying new learning into clinical practice including; delivering education, role modelling, developing new resources and or clinical procedure or information. Examples of role modelling of new behaviour included actively participating in clinical working parties, clinical trials and research, leading by example, engaging in more dementia friendly conversations with colleagues, patients and carers and by delivering more compassionate care to patients.

An analysis of the feedback implies a majority of the projects were reported to be sustained for a period of 12 months or more. Nonetheless our participants also reported reasons for not implementing a CPI activity. Half of the participants cited lack of time and workplace competing demands as barriers. Other factors that played a role were the lack of workplace resources, management support and colleagues, and not feeling in a position to influence change. These findings are confirmed by the fact that smaller proportions of participants reported that their CPI activity had an impact on the workplace (29%) and the system (8%).

An analysis of the literature [20] verifies The Network includes all the features required for an effective online learning environment in that it is: a platform that is self-directed [21, 22], individualised [12, 22], interactive [8, 22], multimodal [8, 22], flexible [2, 23, 24], accessible [2, 23, 24], consistent [8] and cost effective [2, 8]. Whilst at the same time, all programs within The Network nurtures critical thinking within a learning community supported by facilitators. This ultimately encourages translation of learning into effective person-centred practice and to develop a critical approach to decision making [2, 26, 28, 30, 31]. Gagnon et.al [26] conducted a quasi-experimental study and concluded that self-directed educational modules increased nurses’ knowledge and skills in relation to evidence-based practice and that e-learning could be a useful method for continual professional development for nurses. Furthermore, Du and colleagues [30] concluded from their systematic review that web-based education has promising effects in improving learners’ knowledge, skills and self-efficacy in performing nursing skills. Digby et.al [38] strengthens the argument that targeted nurse education must be considered to ensure health systems deliver appropriate person-centred care to people with dementia. Innes and co-workers [31] evaluated student’s views of the delivery modes and learning impact of the first online postgraduate program in dementia studies and found that the majority of respondents (65%) reported their participation in the Dementia Studies program as broadening their thinking, with 61% reporting that it broadened their practice*.* The participant’s perspectives on The Program characteristics (Table 2) is supported by the findings of the thematic analysis of the literature which identified five key themes: *Applicability (88%)*, *Attractiveness (78%), Functionality (59%), Learning interaction (62%) and Implementation into practice (99%)* [23]*.* These key themes also strongly align with Kirkpatrick’s identified core level model [12, 21, 25, 27, 32, 33] to evaluate training and education provision. Nonetheless, it needs to be acknowledged that some participants experienced difficulties with online learning including time limitations, people posting at the same time during chats so comments were missed, and as stated above only a small percentage felt that their implementation activities had impacted their workplace (29%) or the system (8%). When developing the program further, the dementia education program developers should take this into consideration especially in relation to workplace change and system change.

## Study limitations

Although this study suggests the ability to translate person-centred care from the online learning environment to improve person centred care for those living with dementia, there were a number of limitations identified.

The data is comprised of self-reported feedback surveys. The results cannot be compared with objective data nor a control group of what occurred in practice so far as dementia and person- centred knowledge and skills are concerned. Self-reporting allows for bias because the learner may inaccurately report outcomes. The people that took part in the survey are also more likely to be biased to those who enjoyed the course and felt it helped them. An added limitation is the differences between self-perceived and actual performance competence. Constructivism as a learning theory supports the value of self-reported surveys and the impact on clinical practice and behaviour. It is suggested that constructivism impacts on building knowledge and learning being controlled by the learner’s ability to manipulate and process information [35, 36]. The authors suggest that further research would be required to establish and measure these differences.

The 2018 follow up survey demonstrated a considerable reduction of survey participants (*N* = 922) from the original numbers (*N* = 1455). Possible explanations for this may be that by 2018, some of the 2013–2016 participants were automatically un-enrolled due to a configuration setting within the LMS, resulting in a reduction in available contacts. At the same time NSW Health was transferring email addresses to a new email domain which may have caused the survey to be delivered to an old email address. As a result, this left 922 participants to invite for the 2018 follow up survey. Potentially the true response rate could have been higher. There is no way of knowing whether those who remained in the study had the same characteristics, opportunities and barriers for implementing learning into practice than in those who potentially did not receive the survey request.

Finally, designing and developing survey questions proved complex when attempting to elicit the most applicable response in translating learning into practice. The research team found limited publications that evaluated online educational programs designed to improve knowledge and translate learning into clinical practice in dementia care. The identification of 14 key themes that were identified in an integrative literature review [20] that could potentially support successful online learning programs requires further investigation.

These limitations acknowledge that systematic and objective measures are needed to determine whether online learning programs and resource opportunities are helpful with improving dementia knowledge, skills and application to practice.

## Recommendations

Further research is needed to add to the body of evidence to show that online learning can be successfully translated into person-centred dementia care.

Research should be undertaken into non-completion rates for online learning programs to establish obstacles and reasons. This information could be applied to future design and course quality.

Future developers seeking to design and develop new and innovative web-based online learning programs for dementia clinicians could be well inspired by our exemplary program structure and delivery mode. This Program may potentially form the training framework for emerging clinicians and academics to develop the skills and knowledge to deal with a growing population of people living with dementia and improve the quality of life and wellbeing of people living with dementia.

Further effort could be placed on empowering and supporting care staff on how to change systems, procedures and practice and engage management in translating training activities into practice.

## Conclusion

The study results, supported by the literature, suggest that a facilitated online program can be successful in training the dementia care workforce.

Participants perceived the online education program positively influenced their knowledge, skills, behaviour and awareness of dementia care. A considerable number of participants indicated they were able to implement and sustain change in the workplace through implementing CPI activities and transfer their knowledge, and skills to staff, clients and the general public. It was found to be easier to change their own work practices or influence colleagues than change organisational systems and work practices. Further effort could be placed on empowering and supporting care staff on how to change systems, procedures and practice and engage management in translating training activities into practice.

The Dementia Care Competency & Training Network appears to meet many of the themes that constitute a successful and effective online platform, as highlighted in the literature. This multimodal online platform, facilitated by clinical experts, may influence knowledge transfer, skills and behaviour, and has the potential to increase workplace practice improvement activities to potentially improve dementia care.

## Data Availability

All data generated or analysed during this study are included in this published article, however if the datasets are required, they are available from the first author on reasonable request.

## Abbreviations

CASP: Critical Appraisal Skills Programme
CMS: Content Management System
CNC: Clinical Nurse Consultant
CPI: Clinical Practice Improvement
HREC: Human Research Ethics Committee
IPA: International Psychogeriatric Association
LMS: Learning Management System
NSW: New South Wales
The Network: Dementia Care Competency & Training Network
The Program: Positive Approach to the Care of the Older Person with Dementia
TOP 5: Five essential strategies to assist health staff to provide optimal person-centred care and communication
